# Verification procedure for isocentric alignment of proton beams

**DOI:** 10.1120/jacmp.v8i4.2671

**Published:** 2007-10-24

**Authors:** George Ciangaru, James N. Yang, Patrick J. Oliver, Martin Bues, Mengping Zhu, Fumio Nakagawa, Hitoshi Chiba, Shin Nakamura, Hirofumi Yoshino, Mosumi Umezawa, Alfred R. Smith

**Affiliations:** ^1^ Proton Therapy Center, Department of Radiation Physics The University of Texas M.D. Anderson Cancer Center Houston Texas U.S.A.; ^2^ Power Systems Division Hitachi America Houston Texas U.S.A.

**Keywords:** Proton beam therapy, radiation cancer therapy, gantry, isocenter, alignment, film dosimetry, Gafchromic EBT film

## Abstract

We present a technique—based on the Lutz, Winston, and Maleki test used in stereotactic linear accelerator radiosurgery—for verifying whether proton beams are being delivered within the required spatial coincidence with the gantry mechanical isocenter. Our procedure uses a proton beam that is collimated by a circular aperture at its central axis and is then intercepted by a small steel sphere rigidly supported by the patient couch. A laser tracker measurement system and a correction algorithm for couch position assures precise positioning of the steel sphere at the mechanical isocenter of the gantry. A film‐based radiation dosimetry technique, chosen for the good spatial resolution it achieves, records the proton dose distribution for optical image analysis. The optical image obtained presents a circular high‐dose region surrounding a lower‐dose area corresponding to the proton beam absorption by the steel sphere, thereby providing a measure of the beam alignment with the mechanical isocenter. We found the self‐developing Gafchromic EBT film (International Specialty Products, Wayne, NJ) and commercial Epson 10000 XL flatbed scanner (Epson America, Long Beach, CA) to be accurate and efficient tools. The positions of the gantry mechanical and proton beam isocenters, as recorded on film, were clearly identifiable within the scanning resolution used for routine alignment testing (0.17 mm per pixel). The mean displacement of the collimated proton beam from the gantry mechanical isocenter was 0.22±0.1 mm for the gantry positions tested, which was well within the maximum deviation of 0.50 mm accepted at the Proton Therapy Center in Houston.

PACS numbers: 87.53.Xd, 87.53.Oc, 87.56.‐v, 87.66.‐a, 87.56.Fc

## I. INTRODUCTION

A major attraction of proton therapy is that the physics of the proton dose deposition, as reflected by the Bragg depth–dose distribution, offers the theoretical possibility to achieve millimetric accuracy in cancer treatment planning. However, such performance also requires accurate delivery of the proton beam to the target volume, a task of utmost clinical importance for conformal radiation therapy in cancer.

Possible sources of difficulty in achieving good accuracy of beam delivery can include the mechanical motion of the gantry and the patient positioning table around a fixed geometric point in space, called the mechanical isocenter. Mechanical errors in the motion of the heavy gantry and misalignments in the beam delivery system can, if not corrected, result in non‐isocentric relative motion between the delivered beam and the nominal isocenter. This problem is perhaps not very significant for passive wide scattered proton beams, but it is a matter of importance for small‐field treatments and for scanned proton pencil beams.

Maintaining the prescribed spatial coincidence of the proton beam to the gantry mechanical isocenter requires an alignment verification method that is fast and accurate and that can be performed on a regular schedule. Our motivation for developing a procedure for this purpose was the acceptance testing activities occurring at the Proton Therapy Center in Houston (PTC‐H). Our proton isocentric gantries are very large (three stories high) and massive (about 190 tons).

Although the gantries are extremely well engineered by the manufacturer (Hitachi, Tokyo, Japan), their isocenters have a sphere of uncertainty of about 0.5 mm diameter. As a gantry is rotated 360 degrees, the mechanical isocenter moves within a sphere of the stated diameter. Therefore, the mechanical isocenter is gantry angle–dependent, and a lookup table is used to correct for these small errors during quality assurance and patient treatment. The work described in the present paper was part of the acceptance tests of the Hitachi system to confirm that the error corrections were properly applied so that true isocentric movements could be achieved with the patient couch and isocentric gantry.

Briefly, the method that we employed was based on analyzing the two‐dimensional (2D) optical image of the proton beam, which we obtained after it passed through an isocentric steel ball supported by the patient couch. The image was captured on radiographic film placed perpendicular to the beam's central axis. This method is basically an extension to proton therapy equipment of an earlier procedure used in stereotactic linear accelerator (LINAC) radiosurgery by Lutz, Winston, and Maleki.^(1)^

Another reported isocenter alignment test system used for proton therapy by Barkhof et al.,^(2)^ which also evolved from the system of Lutz et al.,^(1)^ uses a scintillation screen viewed by a charged‐coupled device (CCD) camera instead of a radiographic film. Instead of a CCD camera, we chose to use radiographic film, as in the procedure of Lutz et al., because radiographic film is easier to handle and analyze, provides a competitive spatial resolution of the measured proton dose distribution, and is free of problems that can occur when using a CCD camera in a radiation environment containing neutrons.

We believe that our system presents refinements that assure high performance of the isocentric alignment test method for proton therapy equipment. The machining of the various pieces of hardware was performed with very tight tolerances, and the spatial positioning of hardware parts in the experimental setup was performed with a sophisticated laser tracking system. A special software correction algorithm, designed by the manufacturer to assist the Hitachi's patient couch positioning, assured that the steel sphere, firmly attached to the couch, always remained at the gantry isocenter, regardless of gantry angle. The small diameter selected for the aperture and the steel sphere resulted in images that facilitated the analysis, as will be discussed.

## II. MATERIALS AND MEASUREMENTS

### A. Hardware for testing isocentric alignment of proton beams

**Figure 1:**
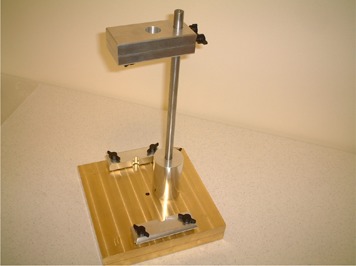
Mechanical jig used for testing the gantry isocenter alignment at the Proton Therapy Center in Houston. The base is a brass plate (4‐cm thickness) that supports the film holder on a 30‐cm metal rod. The brass plate has a central hole (8‐mm diameter), and the film holder has a corresponding hole (20‐mm diameter). This mechanical jig is loaded in the snout.

The mechanical jig that was used to test the proton beam isocentric alignment consisted of a brass plate (4‐cm thickness) that supported a film holder on a 30‐cm metal rod. The brass plate had a central hole (8‐mm diameter), and the film holder had a corresponding hole (20‐mm diameter). Fig. 1 shows this hardware.

**Figure 2:**
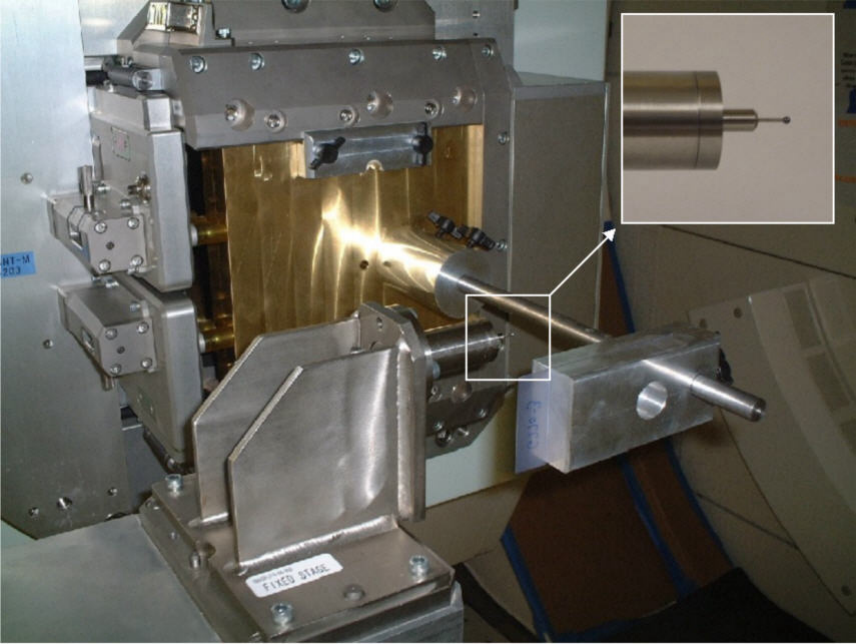
The hardware used to test isocenter alignment with the gantry at 270 degrees. The aperture plate is shown clamped in the snout on the gantry nozzle. The steel sphere (2‐mm diameter) is shown positioned at the gantry mechanical isocenter by the metal holder assembly, which is attached to the patient couch by a metal couch extension. A close‐up of the steel sphere and a portion of the metal holder, which includes a steel rod (1‐mm diameter), is shown in the expanded view (inset).

As Fig. 2 shows, the aperture plate of the mechanical jig was placed in the compensator holder, in the snout of the proton beam delivery nozzle. The mechanical parts were precisely machined to minimize positional uncertainty and to ensure that the testing system would be easy to operate and would fit together with repeatability at each use.

To collimate the proton beam from the passive scattering nozzle to the size of a pencil beam, the circular hole (8‐mm diameter) in the brass aperture plate was centered on the geometric central axis of the beam. The resulting Gaussian‐like cross‐field proton beam distribution had a maximum that was used to identify the center of the collimated proton beam on the film image. We note here that Barkhof et al.^(2)^ used a circular opening 60 mm in diameter in a brass block centered on the geometric beam axis to collimate the proton beam. The result was a near‐flat cross‐field proton distribution, which needed some analysis to identify the center of the collimated proton beam image. Of course, an aperture would not be needed for a proton beam from a scanning nozzle.

The holder for the steel sphere (2‐mm diameter) that was attached to the patient couch with a special metal extension is also visible in Fig. 2. The patient couch was used to position the sphere at the mechanical isocenter of the gantry so that it would intercept the proton beam passing between the holes in the aperture plate and the film holder. This process will be discussed later.

The diameter chosen for the steel sphere (2 mm) is somewhat comparable to that used by Lutz et al.^(1)^ (4.8 mm) for X‐rays, but is much smaller than that used by Barkhof et al.^(2)^ (25.4 mm) for protons. We aimed to keep the size of the sphere as small as practicable so as to facilitate localization of its center on the film image as precisely as possible.

The next two subsections briefly describe the measurements that preceded the actual isocentric alignment tests reported in this paper. These measurements were taken to verify that the aperture hole was aligned with the geometric central axis of the beam and that the steel sphere, supported by the patient couch, was aligned with the mechanical isocenter of the gantry.

### B. Aperture alignment with the beam‐line geometric central axis

The aperture in the brass plate was machined so that its center would coincide with the center of the plate within 0.02 mm. The brass plate fits with a clearance of about 0.12 mm into the slots that usually hold the compensator block in the snout of the beam delivery nozzle. With the nozzle aligned correctly, the center of the aperture should intercept the geometric axis of the beam delivery hardware. We aimed to verify that the portion of the proton beam that passes through the 8‐mm aperture also passes through the gantry isocenter.

To verify that the nozzle alignment was correct, a laser tracker system [Laser Tracker II PLUS and Pro‐X 9.1 software (Automated Precision, Rockville, MD)] was used to make precision measurements (to within 0.025 mm) meant to align the beam aperture with the geometric central axis of the beam line to within 0.2 mm. This uncertainty includes positional uncertainty introduced by extending or retracting the snout and positional uncertainty introduced by gravitational sagging when loading the test jig in the snout at gantry positions other than 0 degrees.

### C. Couch motion automatic alignment with the gantry mechanical isocenter

Displacement precision measurements performed by the manufacturer and our proton physics team showed that the mechanical isocenter of the Hitachi's gantry was contained within a virtual sphere approximately 0.5 mm in diameter. These measurements were used to construct a couch correction table that provides the difference between the geometric (with respect to specified room fiducials) and mechanical isocenters at each gantry angle. The *x‐y‐z* values of the correction table are embedded in the couch controller software. After each gantry rotation, values from the correction table are automatically uploaded to the treatment room pendant and are used to reposition the couch to correct for the non‐isocentricity of the gantry.

We performed measurements with the laser tracker to position the couch so that the center of the steel sphere was located at the mechanical isocenter of the gantry. Because the steel sphere was supported by the couch, the couch correction algorithm permanently kept the steel sphere at the gantry mechanical isocenter.

### D. Measurements

**Figure 3:**
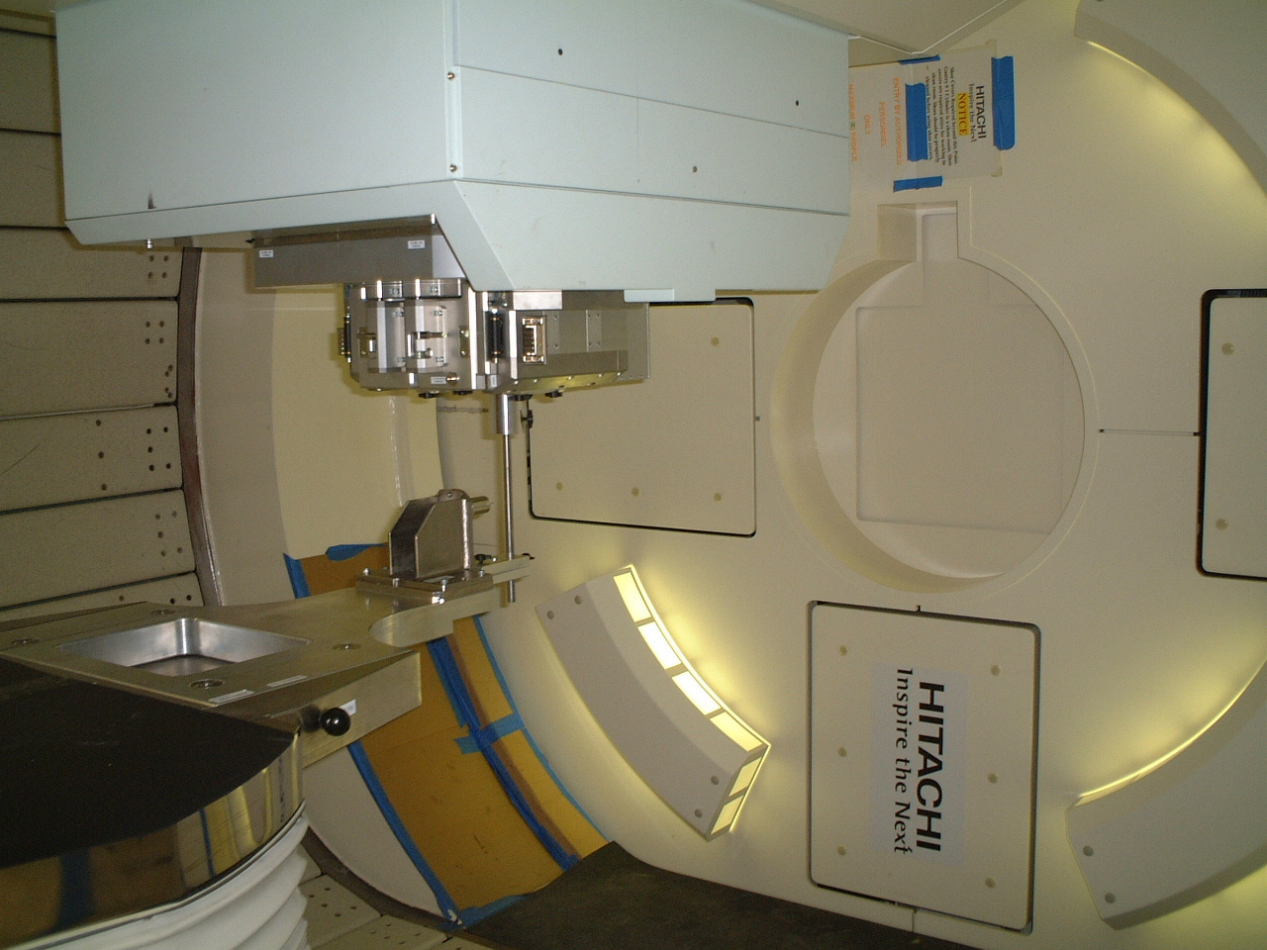
A view of the test setup for the isocenter beam alignment test with the gantry positioned at 0 degrees. The entire metal couch extension that was used to attach the steel sphere (2‐mm diameter) to the patient couch is seen. The beam runs downward through the hole (8‐mm diameter) in the brass plate inserted into the snout, passes through the sphere (2‐mm diameter) and is then intercepted by the EBT film.

As a prerequisite to the measurements, the aperture plate of the mechanical jig was placed in the compensator holder in the snout of the proton beam delivery nozzle, and the center of the steel sphere was secured at the mechanical isocenter of the gantry, as described in earlier subsections. Also, the range modulation wheel for the 120 MeV proton energy selection was installed in position. Two people carried out these operations in about 30 minutes. Measurements could then commence to determine whether the proton beam itself was aligned with the gantry mechanical isocenter. Fig. 3 shows a view of the test hardware setup used for the isocentric beam alignment verification, with the gantry positioned at 0 degrees.

During the measurements, the gantry was rotated, both clockwise and counterclockwise, to each angle to be tested. After each gantry rotation, the treatment room pendant automatically repositioned the couch using the values from the correction table discussed in subsection C.

Before each measurement, a piece of film 5 cm by 10 cm was clamped into the holder, which was held 28 cm from the aperture. The film panels were irradiated perpendicular to the proton beam with doses less than 200 cGy. This low dose level was adequate for our purpose because it produced film images of sufficient quality with reasonably short irradiation times during the tests.

The film that we used was the new self‐developing radiochromic film Gafchromic EBT (International Specialty Products, Wayne, NJ). Although this film has been often used for intensity‐modulated radiation therapy,^(^ ^3^ ^–^ ^8^ ^)^ we are not aware of reports in the literature about its use in proton radiation dosimetry.

The range modulation wheel was stopped with the 120 MeV proton beam incident on the minimum thickness of the wheel. This proton energy was chosen both to prevent penetration by protons through the brass plate installed in the nozzle and to allow enough proton absorption in the steel sphere for achieving a good film image.

The effective time spent by one person to perform the alignment verification procedure for each gantry movement to a particular test angle was usually less than 10 minutes.

### E. Using Gafchromic EBT film and flatbed scanner

Previous studies^(8)^ recommended using a flatbed scanner in transmission mode to obtain optimal results with the Gafchromic EBT type of film. In our study, an Epson 10000 XL scanner set in light transmission detection mode was used to scan the films in 16‐bit grayscale with spatial resolution of 150 dpi (that is, 0.17 mm/pixel).

To obtain the greatest image uniformity possible, the films were centered on the scanner's glass bed. Also, in keeping with the manufacturer's recommended film‐scanning protocol, all film panels were identically directionally positioned on the scanner's bed.

The computer files containing the pixel values corresponding to the detected transmitted light were saved in the tagged image file format (TIFF). Matlab‐based DoseLab software,^(9)^ which was developed in‐house at the M.D. Anderson Cancer Center, was later used to analyze these files.

As is customary in film dosimetry work, we defined the optical density (OD) as the logarithm of the inverse transmittance of the film. DoseLab works with TIFF files that save image data in integers; to gain some resolution, the program scales the OD values by 10 000.

### F. Background correction for the scanned film images

Flatbed scanners such as the model used in the present study use a long, diffuse light source. Scattering of the light can result in output that appears to have been made with a lamp that has poorer light transmission at its ends than at its center. The inherent inconsistency in film density also contributes to the existence of an uneven image background.

We determined the spatial uniformity of the scanner response over the scanning window by measuring transmittal of scanner light through blank (unexposed) films. In general, the scanner uniformity test results, expressed in digitized pixel values of light intensity, presented deviations smaller than 1% from the mean. However, in our tests, the radiation dose level was small, and we considered it necessary to perform background correction for the film images.

The pixel‐by‐pixel method of background correction has been previously described (for example, by Wilcox et al.^(8)^) and is part of the commercial software package FilmQA (3Cognition, Port Jefferson, NY). Before being exposed to radiation, all the blank films were scanned and their OD image TIFF files were created with the DoseLab software. The scanning operation was repeated to obtain the OD image TIFF files of the exposed films. The blank film images were subtracted pixel‐by‐pixel from the corresponding exposed film images.

## III. ANALYSIS AND RESULTS

### A. Proton beam shape and size characterization

**Figure 4:**
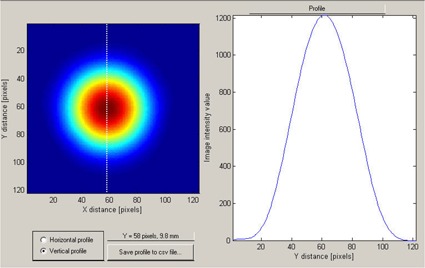
The two‐dimensional optical image (left panel) of the proton beam after it has passed through the aperture (8‐mm diameter) in the brass plate. The dotted line indicates the space direction of a one‐dimensional cut through the *x‐y*‐OD (optical density) matrix, along which the image intensity profile was recorded (right panel).

We performed a preliminary measurement of the proton beam distribution with the range modulation wheel stopped at its minimum thickness, which corresponded to the highestrange 120 MeV protons passing through. The hardware jig for the isocenter alignment testing was attached to the snout on the gantry nozzle so that the beam was passing through the aperture, but for these measurements, the steel sphere was not mounted on the couch. Fig. 4 shows the 2D optical image of the beam obtained from this measurement.

**Figure 5:**
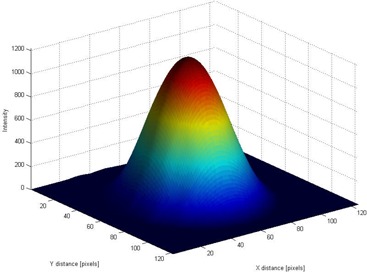
The three‐dimensional film optical image of the beam after it has passed through the isocentricity alignment test system without the steel sphere between the aperture and the film. The third dimension is the optical density; it is color‐coded for visual impact.

Our analysis used OD profiles (Fig. 4, right panel) that were extracted from the beam optical images (Fig. 4, left panel). The proton distribution across the beam was symmetric relative to the beam's central axis, but as could be expected for a beam passed through a relatively narrow aperture,^(10)^ its shape was not that of a pure Gaussian function. The beam's full width at half maximum was 8 mm as determined by the aperture plate mounted on the nozzle, and it is not that of a pure Gaussian with the same maximum height. This effect can also be seen in the three‐dimensional (3D) graphical representation in Fig. 5.

The simulations of Barkhof et al.^(2)^ showed that, for a sphere of 25.4 mm diameter, the beam's collimator diameter should be at least 60 mm (that is, in the ratio of 2.36) so as to minimize distortion effects when the sphere surface approaches the beam's penumbra. In our case, the diameter of the aperture was 8 mm, which was considerably larger than the 2 mm diameter of the steel sphere (that is, their ratio was 4) and the 80% – 20% falloff of the penumbra was approximately 3 mm. During our tests, we never saw the image of the sphere crossing the beam's penumbra. For this reason, the EBT film image was able to clearly capture the geometric relationship between the steel sphere and the aperture in the jig.

### B. Film image data analysis

According to the EBT film manufacturer's protocol, the self‐developing process was considered in practice to be complete 24 hours after exposure to the proton radiation. At that point, the exposed films were scanned and the data saved as TIFF files for later analysis with the DoseLab software tools.

**Figure 6:**
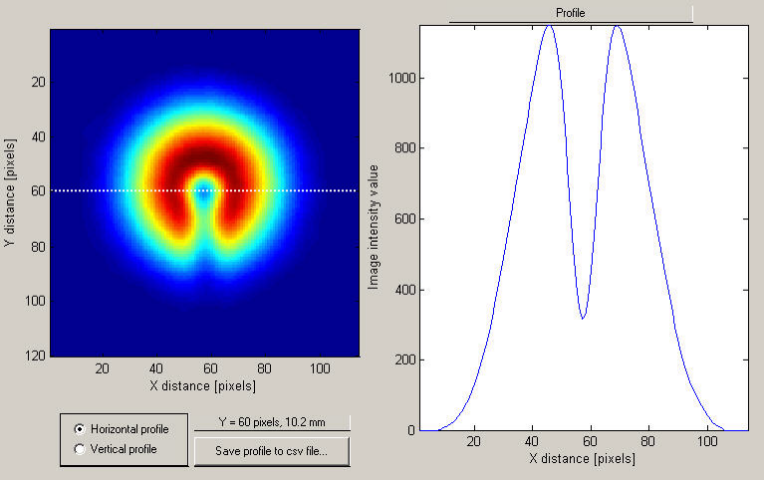
The two‐dimensional film optical image (left panel) of the proton beam after it has passed through the aperture and through the steel sphere positioned at the mechanical isocenter. The dotted line indicates the space direction of a one‐dimensional cut through the *x‐y*‐OD (optical density) matrix, along which the image intensity profile was recorded (right panel).

Fig. 6 shows a 2D optical image of the beam, color‐coded to visually represent OD values. The DoseLab program allows one‐dimensional profiles to be taken in any direction, such as that shown in Fig. 6 (right panel). Of course, the area affected by the presence of the steel rod supporting the isocentric steel sphere had to be avoided, but we did not find that limitation to be serious. The position of the steel sphere is seen to be well delimited because of the good spatial resolution of the film and the smallness of the sphere.

**Figure 7:**
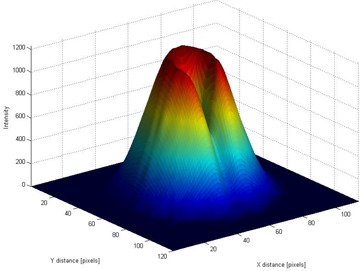
Three‐dimensional film optical image of the beam after it has passed through the isocentricity alignment test system, with the steel sphere (2‐mm diameter) between the beam aperture and the film.

Fig. 7 shows a 3D film optical image of the beam after it has passed through the isocenter alignment test equipment. The effect of the dose absorption by the steel sphere placed at the mechanical isocenter and also by its supporting steel rod can clearly be seen. This representation provides an overall view of where the beam axis may be situated with respect to the mechanical isocenter.

As is customary in film dosimetry work, the OD data were smoothed by passage through a standard averaging Matlab filter that is used by the DoseLab software to improve the robustness of the numerical analysis. The OD profiles taken through the mechanical isocenter—such as that shown in Fig. 6—were then saved and analyzed quantitatively in Excel (Microsoft, Redmond, WA). Extracting the deviation of the proton beam central axis from the mechanical isocenter required only a straightforward numeric analysis. In some cases, when some slight beam misalignment existed, the OD profiles were not as symmetric as was the one shown in Fig. 6. Nevertheless, the data analysis was carried out in the same way for all situations.

**Figure 8:**
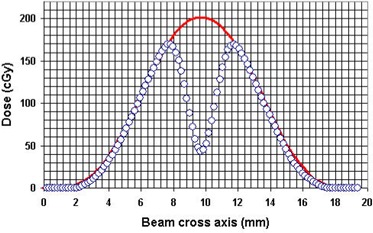
The beam profile film images, taken without (solid line) and with (open circles) the steel sphere installed at the mechanical isocenter, as shown in Figs. 4 and 6 respectively. The beam central axis position corresponds to the maximum of the solid line curve and the steel sphere center position corresponds to the minimum of the circle curve. The optical density data have been converted to dose in this graph, but the conversion was not really required for position analysis.

The center of the steel sphere position, representing the gantry mechanical isocenter, was estimated directly from the graph, because that position was always clearly delimited for the cases that were tested as a minimum in the profile curve, as seen in Fig. 6. Finding the center of the beam's optical image required a somewhat more elaborate operation. The obvious approach would be to place the undisturbed beam penumbra image over the beam penumbra image taken with the sphere and the rod installed, as shown in Fig. 8. However, using that approach would mean obtaining an extra film for the undisturbed beam film radiography at every gantry angle tested, which could become quite tedious.

We verified that the presence of the steel sphere did not in fact alter the shape of the outer shell of the optical image of the beam profile. We could therefore confidently find the center of the beam's optical image from the beam penumbra image obtained with the sphere and the rod installed simply by taking the average of the mean positions of corresponding isodose pairs of points from both sides of the outer beam profile (interpolating between points when necessary). This operation was facilitated by the earlier smoothing of the data to eliminate spurious spikes in the optical image files from the occasional dust grains and marks on the film.

### C. Results

Using the procedure described in the preceding subsection, the centers of the small sphere and of the beam aperture images were found, and their relative distances were measured. The measurements were made for 16 gantry movements to the angles 225, 270, 315, 0, 45, 90, 135, and 180 degrees, in both the clockwise and counterclockwise directions. For each gantry angle, we analyzed three profiles drawn through the mechanical isocenter in the film image, one in the *x* direction and two others in directions rotated by +30 degrees and −30 degrees from the *x* axis of the optical image.

For each measurement, the requirement was that misalignment between the proton beam and the mechanical isocenter should not exceed 0.5 mm. Our measurements showed that, within a general experimental uncertainty of 0.27 mm per individual measurement, the misalignments were between 0 mm and 0.38 mm, consistently well bellow the required limit.

Our tests did not reveal, within the stated uncertainty, misalignments that correlated with any particular gantry angle or direction of rotation. The numerous tests performed in the course of this study showed that the proton beams at the PTC‐H gantries are being directed to within 0.5 mm of the mechanical isocenter target. This finding is reflected in the mean displacement of 0.22±0.1 mm of the proton beam from the gantry mechanical isocenter, found from the stated 16 gantry angle measurements.

## IV. CONCLUSIONS

During acceptance tests conducted at PTC‐H, we developed a testing system to verify the isocentric alignment of proton beam delivery. The testing equipment consists of a mechanical jig that can be attached quickly and precisely by any personnel to the beam delivery nozzle and of a small steel sphere that is installed on the patient couch and that intercepts the proton beam at the gantry mechanical isocenter. An important feature of the testing procedure is the correction algorithm for couch movement, which was used to keep the steel sphere isocentric regardless of gantry position.

Gafchromic EBT film inserted in the jig detected the proton radiation beam. To our knowledge, this brand of film has not yet been used on a significant scale for proton radiography. We found it useful for the work presented here. Some of the attractive features of this film were its convenience in terms of its self‐developing property, its reduced sensitivity to ambient light (which provides freedom to manipulate the film with ease in the experimental area), its optical transparence before radiation exposure (which offers the possibility to scan the blank films so that a pixel‐by‐pixel uniformity correction can be applied to the scanned image of the exposed films), and its ability to be immersed in water for considerable periods of time without damage.

We found that using a small‐diameter steel sphere at the gantry mechanical isocenter, as in the Lutz et al. system,^(1)^ considerably improved the ability of post‐exposure analysis to pinpoint the sphere's position on the optical film image. Relative distance to the central axis of the proton beam could then be determined using fairly straightforward data analysis.

Our isocenter alignment test system proved to be easy and reliable to use. The system appears to have the potential for use both in equipment commissioning and in routine quality assurance.

## ACKNOWLEDGMENTS

We thank Dr. Isaac I. Rosen for help in adapting the DoseLab software package, which was developed under his guidance, to the needs of this study. We also thank Dr. David F. Lewis and other technical personnel at International Specialty Products for their time.

## Supporting information

Supplementary Material Files

Supplementary Material Files
