# Supplementary figures and images for: Verification procedure for isocentric alignment of proton beams
Source: J Appl Clin Med Phys. 2007 Oct 24;8(4):65–75. doi: 10.1120/jacmp.v8i4.2671 (PMC5722618; doi:10.1120/jacmp.v8i4.2671)

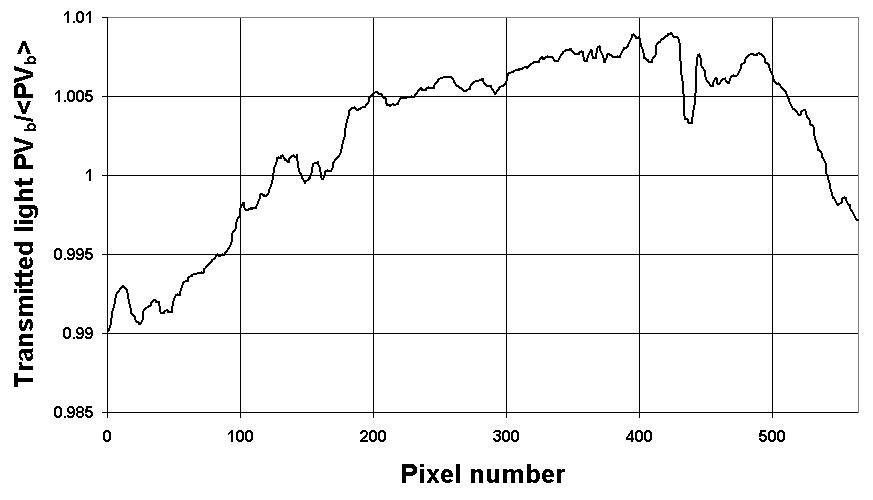

Supplement: Supplementary file 1 — Supplementary Material Files [file ACM2-8-065-s001.jpg]

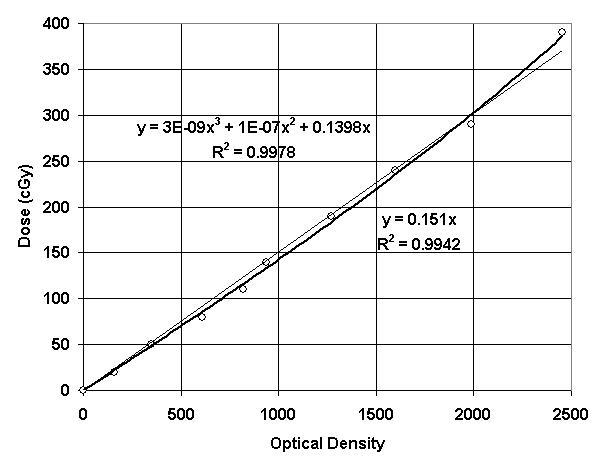

Supplement: Supplementary file 2 — Supplementary Material Files [file ACM2-8-065-s002.jpg]
